# Supplementary material for: RiboTag-based RNA profiling uncovers oligodendroglial lineage-specific inflammation in autoimmune encephalomyelitis: implications for pathogenesis
Source: J Neuroinflammation. 2025 May 21;22:135. doi: 10.1186/s12974-025-03463-x (PMC12093676; doi:10.1186/s12974-025-03463-x)
Supplement: Supplementary file 1 — Supplementary Material 1 [file 12974_2025_3463_MOESM1_ESM.docx]

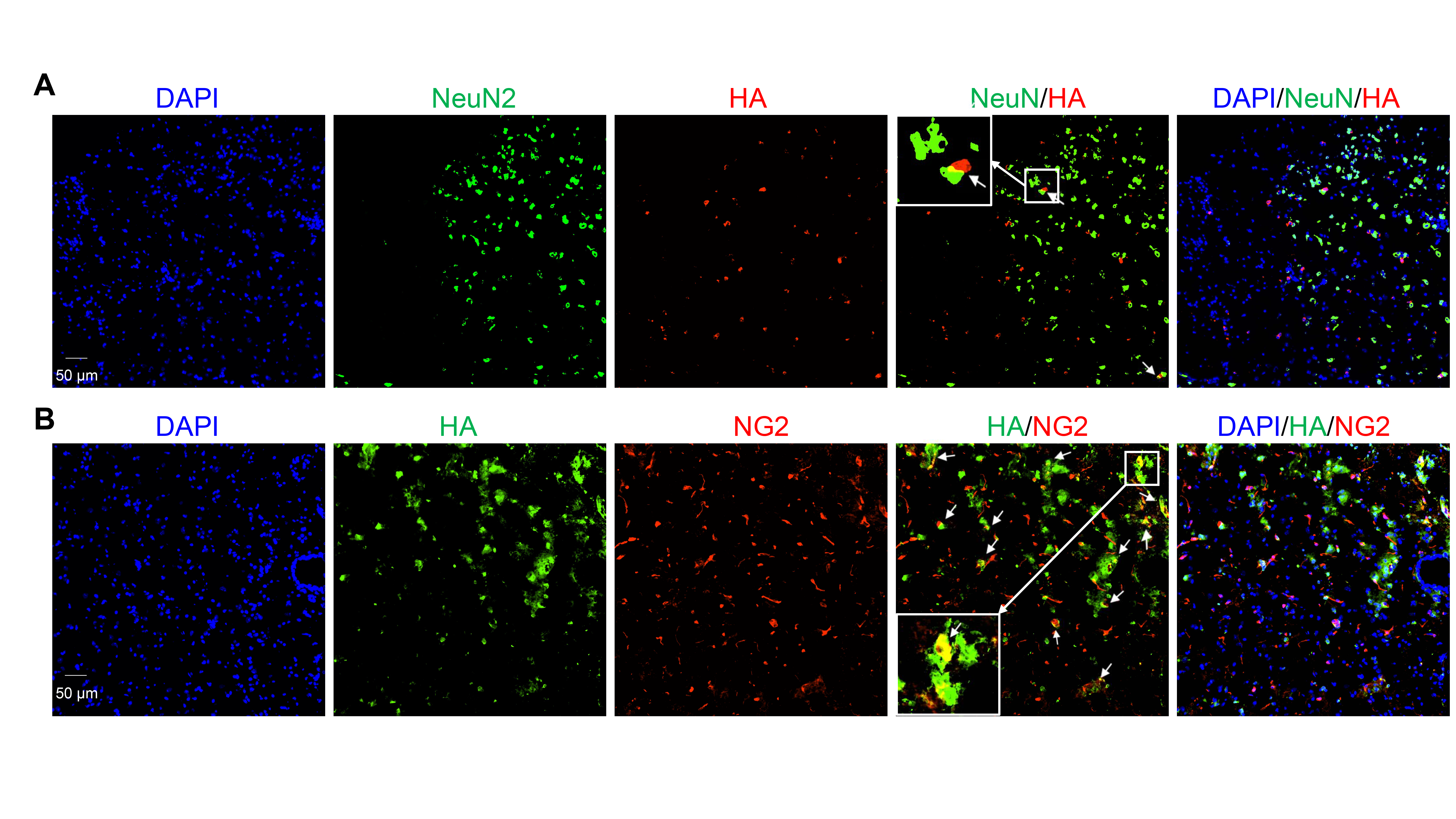


**Supplemental Figure 1. Fluorescent immunostaining on spinal cords of Olig2-Cre RiboTag mice**

(A-B) Fluorescent immunostaining was performed on spinal cords obtained from Oligo2-Cre RiboTag mice using specified antibodies, as indicated. Co-localization is visualized in yellow following image merge, indicated by arrows, with nuclei counterstained using DAPI. Related to Figure 1A.


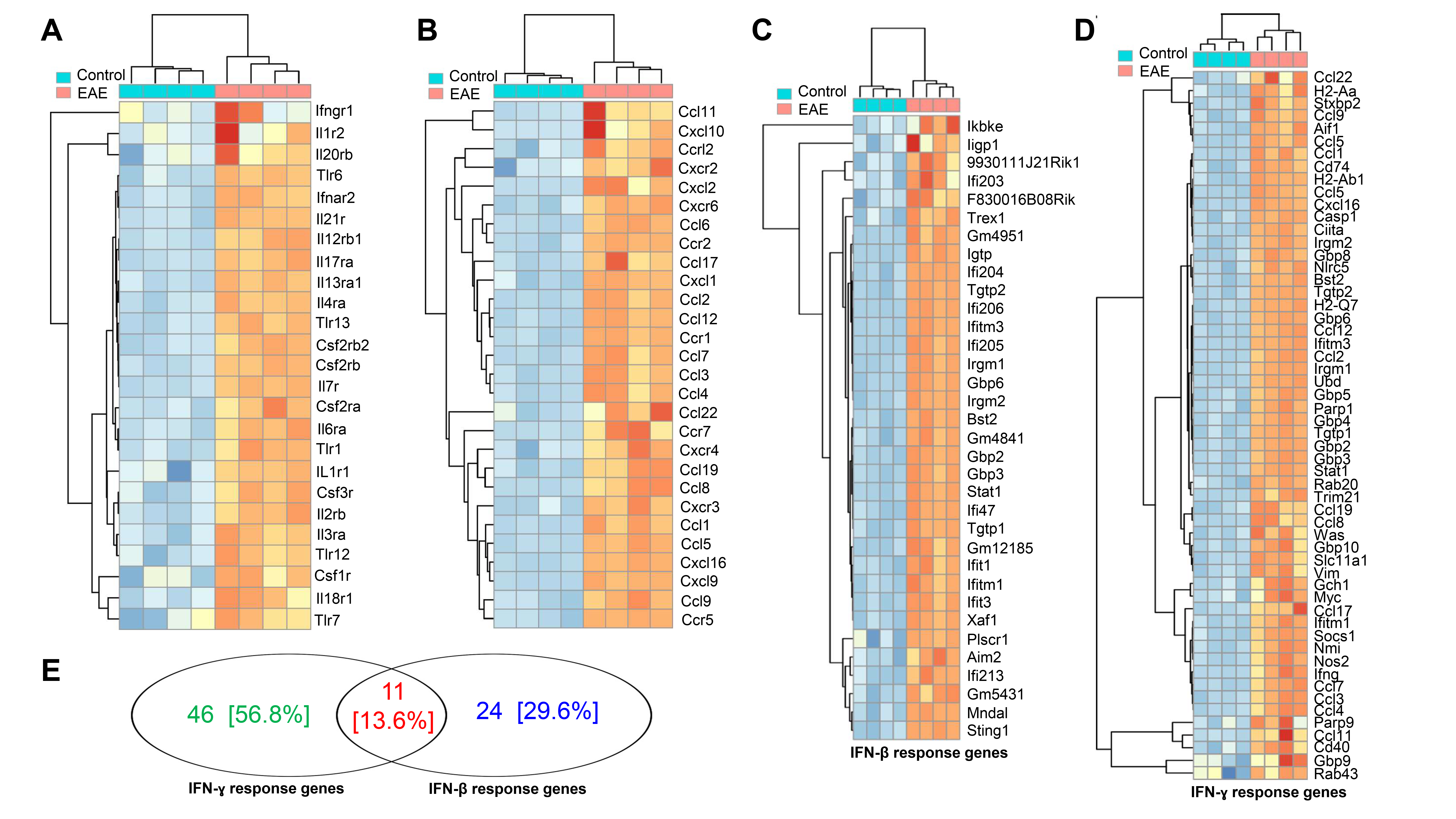


**Supplemental Figure 2. Heatmap illustration of differentially expressed genes**

Spinal cords from Oligo2-Cre RiboTag EAE mice at disease onset and naïve control mice were homogenized, and mRNA was isolated via immunoprecipitation with anti-HA antibodies targeting ribosomes. The purified mRNAs underwent RNA-Seq using an Illumina NovaSeq sequencer, and the resulting data were analyzed. The heatmap depicts differentially expressed genes categorized as follows:

(A) Cytokine receptors and Toll-like receptors (Tlrs).

(B) Chemokine and chemokine receptors.

(C) IFN-β response genes.

(D) IFN-γ response genes. Within the GO biological process category (Panel A-D).

(E) Venn diagram of IFN-γ and IFN-β response genes. n = 4/group. Related to Figure 2.

**Supplementary Figure 3. Change of oligodendroglia in the Ifngr1^fl/fl^ mice and Olig2-Cre Ifngr1^fl/fl^ mice before and after EAE.**


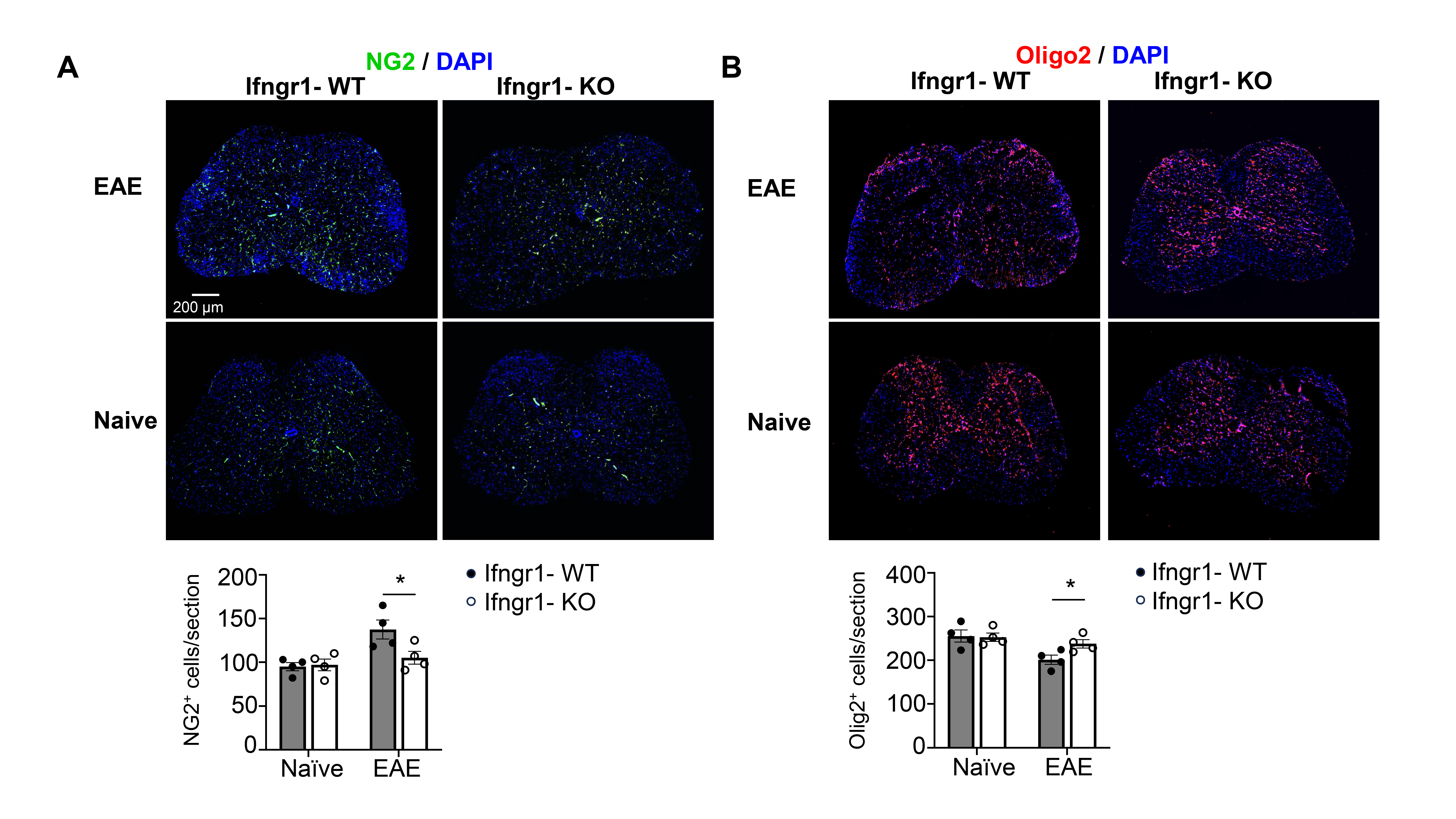


A). NG2^+^ cells per section were counted from lumber spinal cords of Ifngr ^fl/fl^ and Olig2 Cre Ifngr ^fl/fl^ mice before and after EAE.

B). Olig2^+^ cells per section were counted from lumber spinal cords of Ifngr ^fl/fl^ and Olig2 Cre Ifngr ^fl/fl^ mice before and after EAE. N=4/group, results are shown as mean ± SEM, * p<0.05. Related to Figure 5.
